# Paralysie sciatique suite à un descellement post-traumatique d'une prothèse de la hanche

**DOI:** 10.11604/pamj.2015.22.385.8613

**Published:** 2015-12-29

**Authors:** Mohamed Amine Karabila, Mohamed Saleh Berrada

**Affiliations:** 1 Service de Chirurgie Traumato-Orthopédie, CHU Ibn Sina, Rabat, Maroc

**Keywords:** Paralysie, descellement, hanche, paralysis, loosening, hip

## Image en medicine

Nous rapportons le cas d'une patiente âgée de 71 ans, victime d'une chute de sa hauteur sur sa hanche droite et dont l'examen clinique objective en plus de la douleur de la hanche d'une hypoesthésie de la face dorsale du pied droit avec déficit de la dorsiflexion. Le bilan radiologique montre une fracture trochantéro-diaphysaire complexe avec un descellement important de la tige fémorale qui est presque extériorisée du canal fémoral (A). La patiente a bénéficié à j+1, d'une ablation de la tige fémorale descellée, reconstruction de la fracture par des câbles et neurolyse du nerf sciatique comprimé par la tige puis reprise par une tige longue verrouillée et un cotyle cimenté (B). A 14 mois de recul (C), la patiente marchait avec une canne avec persistance d'un déficit des releveurs des orteils 3/5 et des fibulaires latéraux 3/5.

**Figure 1:**
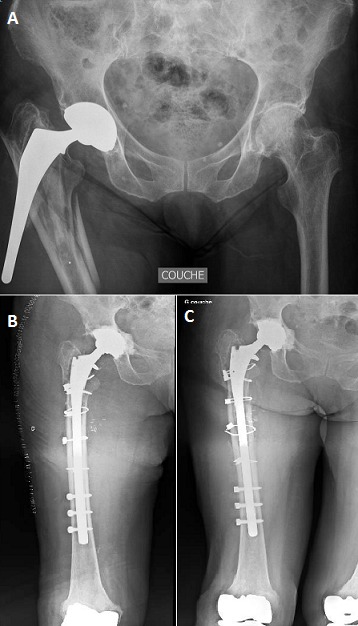
A): radiographie du bassin montrant le descellement important de la tige suite à la fracture de fémur ; B): contrôle post opératoire immédiat de la prothèse de reprise (tige fémorale et cotyle) avec la bonne réduction de la fracture fémur :C) : contrôle après 6 mois montrant la consolidation et la bonne prise de la prothèse

